# Anodic and Mechanical Behavior of Carbon Fiber Reinforced Polymer as a Dual-Functional Material in Chloride-Contaminated Concrete

**DOI:** 10.3390/ma13010222

**Published:** 2020-01-04

**Authors:** Liangliang Wei, Ji-Hua Zhu, Zhijun Dong, Jun Liu, Wei Liu, Meini Su, Feng Xing

**Affiliations:** 1 Guangdong Province Key Laboratory of Durability for Marine Civil Engineering, College of Civil and Transportation Engineering, Shenzhen University, Shenzhen 518060, China; weiliangliang@email.szu.edu.cn (L.W.); liujun@szu.edu.cn (J.L.); liuwei@szu.edu.cn (W.L.); xingf@szu.edu.cn (F.X.); 2 School of Traffic and Environment, Shenzhen Institute of Information Technology, Shenzhen 518172, China; dongzj@sziit.edu.cn; 3 School of Mechanical, Aerospace and Civil Engineering, University of Manchester, Manchester M1 7JR, UK; meini.su@manchester.ac.uk

**Keywords:** CFRP, impressed current cathodic protection, dual-functional, chloride, tensile strength

## Abstract

Carbon fiber reinforced polymer (CFRP) has been used as a dual-functional material in a hybrid intervention system (ICCP-SS) which integrates the impressed current cathodic protection (ICCP) and structural strengthening (SS). The mechanical behavior of CFRP as an anode has been investigated in some solution environments. However, the anodic and mechanical behavior of CFRP bonded to concrete is unclear. This paper focuses on the anodic and mechanical performance of CFRP bonded to the chloride-contaminated concrete by conducting an electrochemical (EC) test. The method of bonding the CFRP to the concrete and the shape of the steel embedded in the concrete were considered. The current densities of 20 mA/m^2^ and 100 mA/m^2^ were applied during 120-day and 310-day EC tests. The electrode potentials and driving voltages were recorded, and the bond interfaces of the CFRP were inspected after EC test. The residual tensile strength and failure modes of the CFRP were analyzed after tensile tests. Finally, the long-term performance of CFRP as a dual-functional material in ICCP-SS system was discussed. Results show that the externally bonding CFRP in ICCP-SS system can not only protect the steel in chloride-contaminated concrete effectively but also maintain 70% of the original tensile strength of CFRP at a charge density of 744 A·h/m^2^. The expected service period of CFRP as a dual-functional material bonded to the chloride-contaminated concrete was determined to be more than 42.5 years.

## 1. Introduction

In recent years, a new intervention system, integrating impressed current cathodic protection (ICCP) with structural strengthening (SS), has been proposed to be ICCP-SS for improving the maintenance of concrete suffered from the corrosion of steels induced by chloride [1,2,3,4]. Structural strengthening by FRP or steel plates has been proven to be an effective method of improving the structural performance of corroded concrete structures, although the performance might degrade later due to the continuous corrosion of the steel in the concrete [5]. However, it is well known that impressed current cathodic protection can effectively mitigate the corrosion of steel by utilizing an electrochemical mechanism [6]. Without structural strengthening, deteriorated concrete structures cannot be recovered to their design capacities. The ICCP-SS was activated by using a dual-functional carbon fiber reinforced polymer (CFRP) material. The CFRP is used as a strengthening material and an anode material. Zhu et al. [1] and Su et al. [3] found that the ICCP-SS system could work effectively in protecting the steel reinforcement and increasing the ultimate strength of concrete columns and beams contaminated by chloride. However, the anodic and mechanical behavior of CFRP suffered from polarization in ICCP was not clarified and requires further investigation.

Some researchers [7,8] have conducted electrochemical (EC) tests of CFRP in aqueous solutions using a current density greater than 2 A/m^2^ for a few months. The EC test is carried out in the laboratory conditions that are intended to simulate the conditions present when cathodic protection current discharges from the anode in a working ICCP system. Sun et al. [7] evaluated the tensile strength of CFRP after being subjected to polarization in 3% NaCl electrolyte solutions. The results showed that the tensile strength of CFRP decreased with increasing charge density. The CFRP in 3% NaCl electrolyte solutions maintained approximately 92% of the tensile strength of the CFRP at a charge density of approximately 555 A·h/m^2^, respectively. Furthermore, Zhu et al. [8] also conducted EC tests of CFRP in simulated seawater with different chloride contents. However, practical concrete conditions are more complex than the stable solution condition. Therefore, the anodic and mechanical behavior of CFRP bonded to the chloride-contaminated concrete must be clarified.

The objectives of this study are firstly to verify the anodic behavior of CFRP in chloride-contaminated concrete; secondly, to evaluate the mechanical behavior of CFRP suffered from anodic polarization in ICCP; and thirdly, to investigate the optimal CFRP bonding method as the dual-functional material in chloride-contaminated concrete. The electrode potentials and driving voltages were recorded during the EC tests. Tensile tests of CFRP were conducted after the EC tests. The failure modes of the CFRP were recorded, and the residual tensile strength was analyzed. Finally, the degradation mechanism and the long-term performance of CFRP as a dual-functional material in ICCP-SS system was discussed.

## 2. Experimental Program

### 2.1. Test Specimens

Standard quartz sand and simulated seawater [9] were used to prepare chloride-contaminated RC specimens. The ratio of chloride ions to water was 1.94% in the simulated seawater. The mix proportion of the chloride-contaminated concrete is given in Table 1. The grade of ordinary Portland cement was 42.5R. The water-to-cement ratio of concrete specimens was 0.37 that is one of the common adopted proportion, and the ratio of sand to cement was 2.0. The grades of standard quartz sand were acceptable according to the requirement of BS EN 196-1 [10], as shown in Table 2. Therefore, the chloride content with respect to the cement weight in concrete was 0.72%. This content is seven times the chloride threshold of 0.1% stated by international guidelines [11,12,13]. All concrete specimens were moist-cured at an ambient environment of 95% humidity and 20 ± 2 °C temperature for 28 days. The 28-day compressive strength of concrete in a size of 100 × 100 × 100 mm was found to be 52 MPa by using the model YAW4206 MTS Servo-hydraulic Compressional Testing Machine.

**Figure 1:**
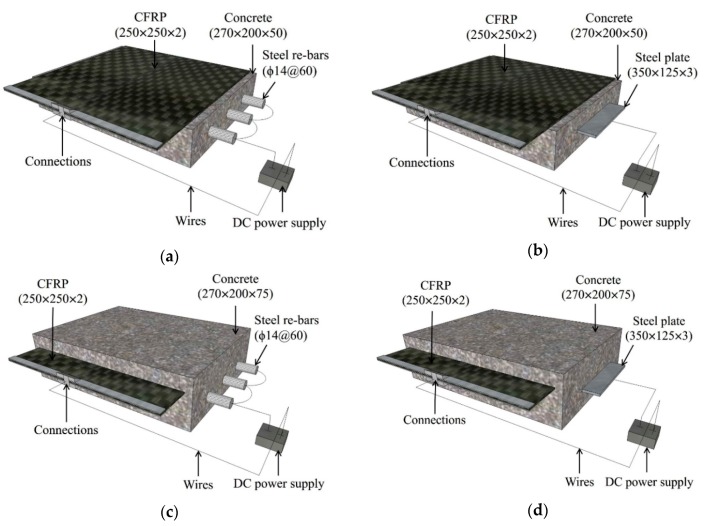
Schematic diagrams of the chloride-contaminated concrete test specimens with bonded carbon fiber reinforced polymer (CFRP) and embedded steel (all dimensions are in mm): (**a**) Steel rebars—externally bonded CFRP (B-EB specimens); (**b**) Steel plate—externally bonded CFRP (P-EB specimens); (**c**) Steel rebars—internally bonded CFRP (B-IB specimens); (**d**) Steel plate—internally bonded CFRP (P-IB specimens).

The multilayer CFRP used in this study had a 60% carbon fiber volume fraction. First, two CFRP bonding methods were investigated: externally bonded (EB) CFRP, which is used to strengthen existing RC structures, and internally bonded (IB) CFRP, which is used during the creation of a new structure. The EB CFRP was adhered to the concrete by an adhesive binder, instead of epoxy resin, mixed with short-chopped carbon fibers [14,15], whereas the IB CFRP was inserted into the concrete during casting. The concrete specimens with EB CFRP are shown in Figure 1a,b, and those with IB CFRP are shown in Figure 1c,d, where one sample was prepared for each concrete specimen. The bonding area (*A_CFRP_*) between the CFRP and the concrete is 500 cm^2^ (i.e., 200 mm × 250 mm). Second, the shapes of the steel embedded in the concrete, including the steel rebars and steel plates, were considered. Three deformed steel rebars with a diameter of 14 mm at a spacing of 60 mm were used for concrete reinforcement, as shown in Figure 1a,c. The yielding strength and ultimate strength of steel rebar were 480 MPa and 602 MPa, respectively. One hot-rolled steel plate with a thickness of 3 mm and a width of 125 mm was embedded in the concrete, as shown in Figure 1b,d. Both ends of the steel rebars and the edge of the steel plate were insulated by epoxy resin; the contact areas of the steel and concrete were 264 cm^2^ (*A_re-bars_*) and 512 cm^2^ (*A_plate_*).

The test specimens subjected to polarization in ICCP were divided into four series with respect to both the CFRP bonding methods and steel shapes, as shown in Figure 1. Table 3 describes all the test specimens; in this table, B indicates steel rebars, P indicates steel plate, EB indicates externally bonded CFRP, IB indicates internally bonded CFRP, and the last number represents the current densities during ICCP in mA/m^2^. RF1 and RF2 are the reference specimens cast by simulated seawater, whereas RF3 and RF4 are cast with deionized water without chloride. The preparation of reference specimens without CFRP are carried out to compare the corrosion conditions of the steel rebars and steel plates with those of other test specimens.

### 2.2. EC Tests Procedure

**Figure 2:**
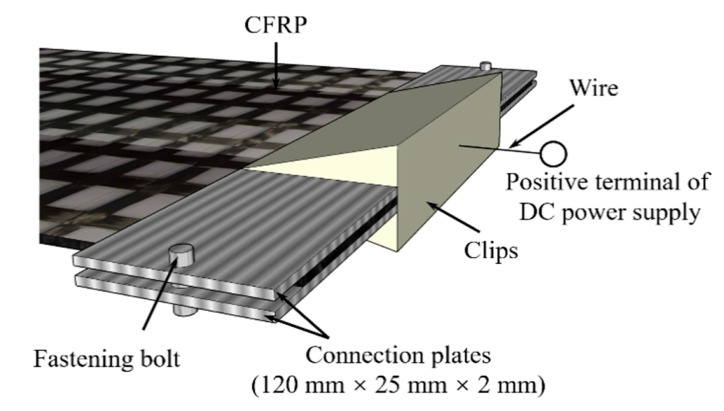
Details of the connection at the CFRP.

The EC tests were performed by applying constant currents from the model LPS605D LodeStar DC power supply (Shenzhen, China), following [16]; the CFRP was used as the anode, and the steel rebars and steel plate were used as the cathode, as shown in Figure 1. In this study, the wires were welded to the steel rebars, and the completed thermite welds were coated. For the connection between the CFRP and power supply positive cable, the CFRP was embedded between two connection plates by bolts, and the power supply cable was then connected to the plates by a clip (see Figure 2). This metal-to-metal connection ensured that the cathode network was electrically continuous. The quality of all connections was verified.

The current density in the ICCP system is a critical factor. A higher current density, which was greater than 2000 mA/m^2^, could be used to perform accelerated EC tests in an aqueous solution over a short test period [7,8]. Although the maximum applied current density in concrete was normally limited to 20 mA/m^2^, the current density may be increased to 50 mA/m^2^ in extreme environments, such as high chloride contamination, moist, wetting and drying, and severe corrosion of steel with a thin concrete cover [17]. Considering the influence of the high current density on the concrete and the cost of testing, current densities of 20 mA/m^2^ and 100 mA/m^2^ were adopted to perform the EC tests for 120 days and 310 days.

### 2.3. Testing Program

During the 120-day testing period, the open circuit potentials (OCPs) were monitored at an interval of approximately two weeks to assess the corrosion state of the steel rebars and steel plate in the chloride-contaminated concrete. In addition, the corrosion rates of the steel were measured at an interval of approximately one month through linear polarization resistance (LPR) measurement by a Model 283 Princeton Electrochemical Workstation (Princeton, NJ, USA). Each concrete specimen in the EC tests procedure was used to perform the LPR measurements that were using a normal three-electrode system. A stable reference electrode (saturated calomel electrode, SCE) was used to measure the corrosion potentials of the steel in the concrete. The CFRP was used as the auxiliary electrode for measuring the current flow (Δ*I*), which was induced by an externally imposed potential shift (Δ*E*) of 20 mV. Thus, the polarization resistance *R_p_* and corrosion current (*I_corr_*) can be calculated with Equations (1) and (2). The value of *B* is normally assumed to be 25 mV for actively corroded steel in concrete [18]. Accordingly, the corrosion current density (*i_corr_*), which represents the corrosion rate, can be obtained between the corrosion current and the steel area in concrete using Equation (3).

(1) $$R_{p}=\frac{\Delta E}{\Delta I},$$

(2) $$I_{corr}=\frac{B}{R_{p}},$$

(3) $$i_{corr}=\frac{I_{corr}}{A_{s}},$$

where *R_p_* is the polarization resistance of steel, Δ*E* is the potential shift of 20 mV during the LPR measurements, Δ*I* is the measured current induced by the potential shift, *I_corr_* is the corrosion current of steel, *B* is a constant value related to the corrosion state of steel and is assumed to be 25 mV for actively corroded steel in concrete, *i_corr_* is the corrosion current density representing the rate of steel corrosion, and *A_s_* is the area of steel bonded with concrete.

During the 310-day EC tests procedure, the driving voltages and 4-h decay potentials were recorded by using Model LR8402-21 HIOKI Memory HiLogger (Hioki, Japan) at an interval of approximately two weeks. The driving voltages between the steel reinforcements and bonded CFRP were measured. The potential measurement was conducted in accordance with [19], in which an SCE in contact with the concrete surface was used to measure the potentials of the steel in the concrete. The instant-off potential (*E_instant-off_*), excluding the internal resistance (IR) drop, and decayed-off potential (*E_decayed_*) were recorded by the same datalogger. A potential decay over 4 h (Δ*E_4-hour_*) could be obtained using Equation (4).

(4) $$\Delta E_{4-hour}=\left|E_{instant-off}-E_{decayed}\right|,$$

where Δ*E_4-hour_* is the potential decay of steel over 4 h, *E_instant-off_* is the instant-off potential of steel excluding IR drop for switching off the external current, and *E_decayed_* is the decayed-off potential after switching off the external current for 4 h.

**Figure 3:**
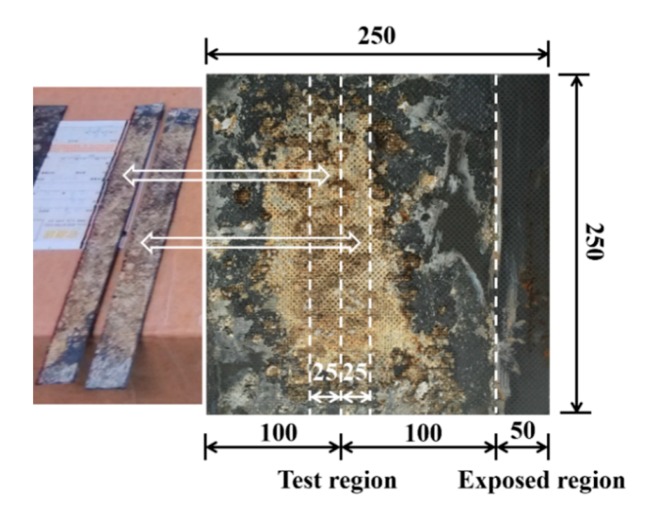
Preparation of the CFRP coupons for the tensile tests after electrochemical (EC) tests (all dimensions are in mm).

When the EC test period was finished, the bonded CFRP was removed from the concrete, and the surface of the concrete was sprayed with a 0.5% phenolphthalein indicator. A color change at the surface of the chloride-contaminated concrete was observed. Finally, the CFRP subjected to polarization in EC test was cut into the specified tensile coupons shown in Figure 3. The dimensions of the coupons were 250 mm × 25 mm × 2 mm. According to [20], the tensile tests of the CFRP coupons were carried out at a displacement rate of 0.1 mm/min by a universal testing machine MTS Model E45. Two samples were prepared. The failure modes were observed and failure loads were recorded by Model DH3820 DongHua datalogger (Jingjiang, China).

## 3. Results and Discussion

### 3.1. Corrosion of Steel

**Figure 4:**
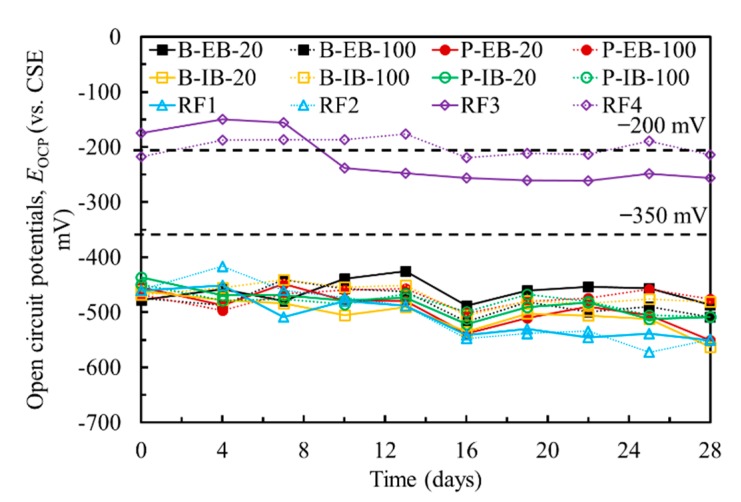
Open circuit potentials of steel in all the specimens during the 28-day curing period.

Figure 4 shows the OCPs of the steel in all the specimens during the 28-day curing period. The OCPs of all the specimens are less than −400 mV, except for the reference specimens RF3 and RF4, which have OCPs of approximately −200 mV. According to [19], if the OCP over an area is more negative than −350 mV (vs. copper sulphate electrode (CSE)), it is >90% likely that reinforcing steel corrosion is occurring. In contrast, the probability of corrosion is small when the OCP is more positive than −200 mV (vs. CSE). Therefore, the steel rebars and steel plate in the chloride-contaminated concrete are corroded at the time of measurement.

**Figure 5:**
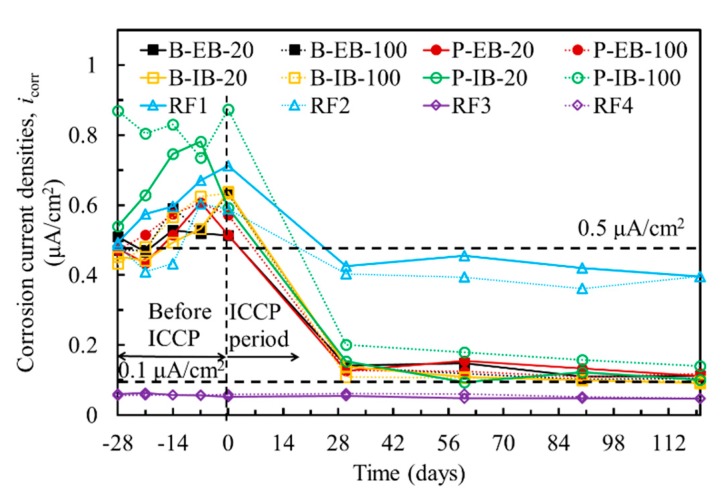
Corrosion current densities of the steel rebars and steel plate before and after EC tests.

In addition to the probabilistic assessment of the corrosion of steel, the corrosion rate can be obtained by measuring the corrosion current densities. Figure 5 shows the corrosion current densities of the steel in all the specimens. LPR measurements were performed before EC tests during the 28-day curing period in which the corrosion current densities of the steel in the chloride-contaminated concrete were higher than 0.5 μA/cm^2^, while specimens RF3 and RF4 in the normal concrete had corrosion current densities smaller than 0.1 μA/cm^2^. Grantham [21] found that reinforcing steel was in the passivated state when its corrosion current density was less than 0.1 μA/cm^2^, while it was in the moderately corroded state when the corrosion current density ranged from 0.5 to 1.0 μA/cm^2^. Considering both the OCPs and corrosion current densities of the steel in the concrete, both the steel rebars and steel plate in the chloride-contaminated concrete were moderately corroded.

### 3.2. Cathodic Protection by Using CFRP

**Figure 6:**
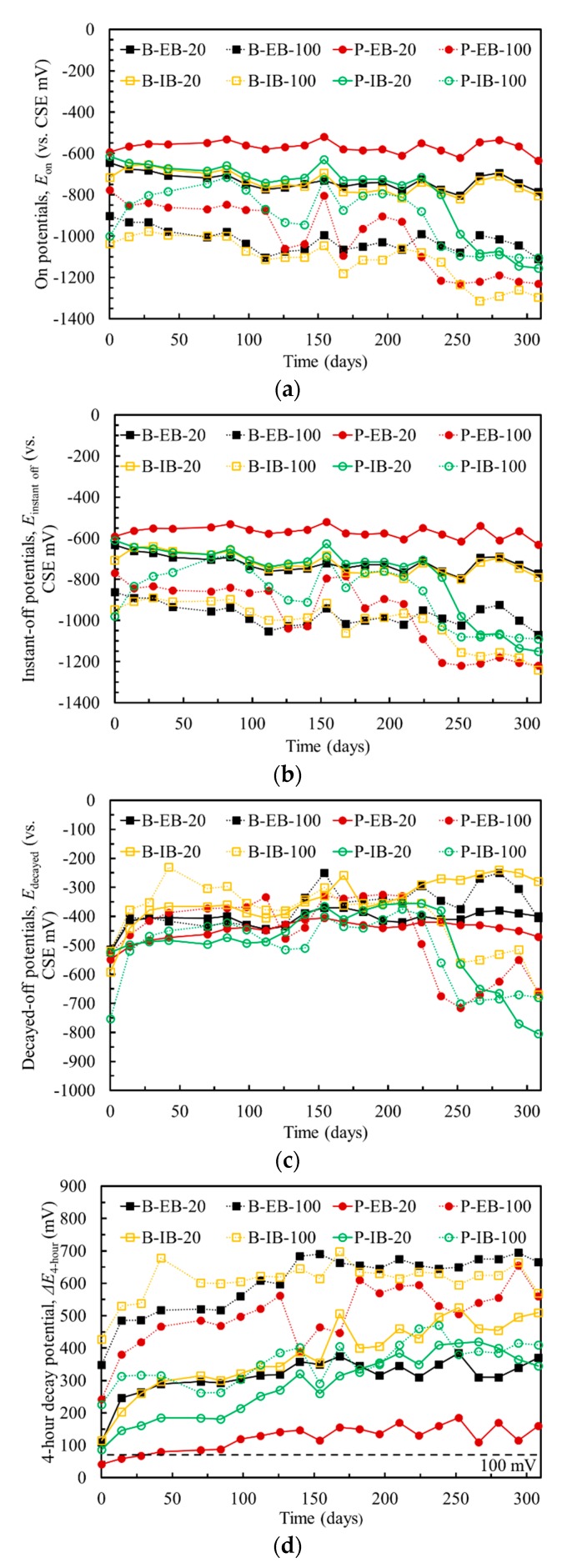
Potentials of the steel rebars and steel plate in the chloride-contaminated concrete before and during EC tests: (**a**) On potentials; (**b**) Instant-off potentials; (**c**) Decayed-off potentials; (**d**) 4-h decay potentials.

To understand the cathodic protection by using CFRP, comprehensive electrochemical signals, such as on potentials, instant-off potentials, decayed-off potentials and 4-h decay potentials, were recorded during the EC tests period. Figure 6a shows the on potentials of the steel in the chloride-contaminated concrete because the direct current (DC) power supply was switched on. The on potentials of the steel gradually shifted to more negative values. Note that the corrosion rate of the steel could be reduced if the steel was in the passivity zone. However, the on potentials of the steel were influenced by the IR of the concrete electrolyte. Generally, the instant-off potentials are used to assess the tendency of corrosion in steel. The instant-off potentials were measured between 0.1 s and 1 s after switching off the DC circuit to prevent the effect of IR drop [22]. Figure 6b shows the instant-off potentials of the steel in the concrete. The results show that RF1 and RF2, which were chloride-contaminated concrete specimens without EC tests, still suffered a relatively high corrosion level, while the corrosion probability of the steel rebars and steel plate in the chloride-contaminated concrete was reduced significantly due to the protection of the ICCP system.

Moreover, an acceptable method to measure the effectiveness of ICCP is to obtain the 100-mV decay as specified by [23]. A minimum of 100 mV of polarization should be achieved by the steel. Figure 6c shows the decayed-off potentials of the steel, which were measured after switching off the power for 4 h; the decayed-off potentials were more negative than −200 mV. Subsequently, the 4-h decay potentials should be obtained according to Equation (4) using the results of the instant-off potentials shown in Figure 6b and decayed-off potentials shown in Figure 6c. The results of the 4-h decay potentials shown in Figure 6d indicate that the minimum 100 mV decay criterion was satisfied. Therefore, the corrosion rate of the steel rebars and steel plate in the chloride-contaminated concrete could be reduced to a negligible level by ICCP.

Furthermore, LPR measurements were performed after a specified polarization time during the EC test period. Figure 5 shows the changes in the corrosion current densities of the steel rebars and steel plates before and during EC tests. The results clearly show that the corrosion current densities decreased considerably by the application of cathodic protection by using CFRP. This result was confirmed by the abovementioned electrochemical signals. All the results validated that the CFRP can be efficiently used as anode in ICCP for the protection of steel in the chloride-contaminated concrete.

### 3.3. Degradation Induced by Cathodic Protection

**Figure 7:**
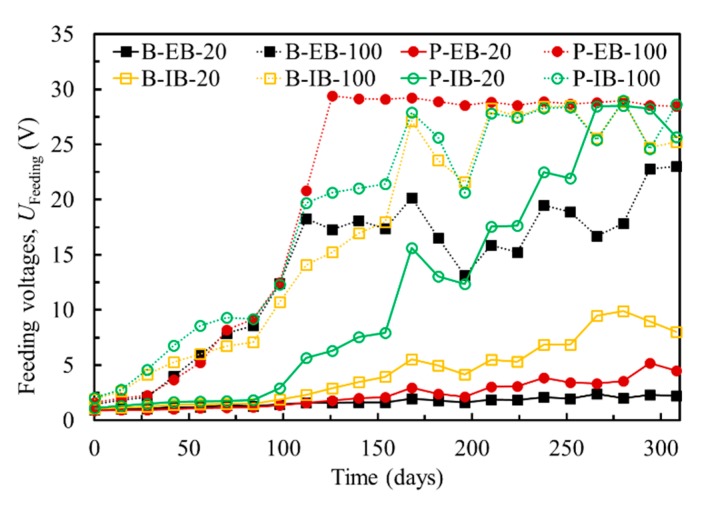
Driving voltages between the steel and CFRP.

The ICCP system must be carefully applied to concrete because it is possible to induce some degradation of the CFRP and bond interface. Figure 7 shows the evolution of driving voltages measured between the steel reinforcements and the CFRP with respect to the test duration. The driving voltages generally increased over time. For the B-EB-20 and P-EB-20 specimens, the magnitude of increase was slight, wherein the driving voltage increased from approximately 2 V at the beginning to less than 5 V at the end of EC test for 310 days. For the B-IB-20 and P-IB-20 specimens, the driving voltages increased significantly after testing for 100 days. For the specimens at a current density of 100 mA/m^2^, the driving voltages increased rapidly because of the high current density used in EC test. The possible degradation of the bond interface and CFRP induced by polarization in ICCP could be presented by monitoring of the driving voltages.

#### 3.3.1. Degradation of Anode Interface

When the protection current transfers from the positive to negative terminal of the DC power supply through the CFRP, concrete electrolyte and steel cathode, electrolytic reactions of water could occur at the anode interface. Due to the inherent alkalinity of concrete, the anodic reaction could be a reduction in OH^−^, as shown in Equation (5). As the OH^−^ at the anode interface is gradually consumed, an acidic environment is developed, resulting in the reduction reaction of H_2_O, as shown in Equation (6). A large amount of H^+^ could be produced at the anode interface, leading to acidification. Moreover, in the presence of chloride in the concrete, the reactions shown in Equations (7) and (8) could also occur, thereby accelerating the acidification at the anode interface.

(5) $${4\;\mathrm{OH}}^{-}\;-{\;4\;e}^{-}\;\to \;2\;H_{2}O\;+\;O_{2},$$

(6) $${2\;H}_{2}O\;-\;4{\;e}^{-}\;\to \;4\;H^{+}\;+\;O_{2},$$

(7) $$2\;Cl^{-}\;-\;2{\;e}^{-}\;\to \;Cl_{2},$$

(8) $$Cl_{2}\;+\;H_{2}O\;\to \;2\;H^{+}\;+\;Cl^{-}+ClO^{-},$$

**Figure 8:**
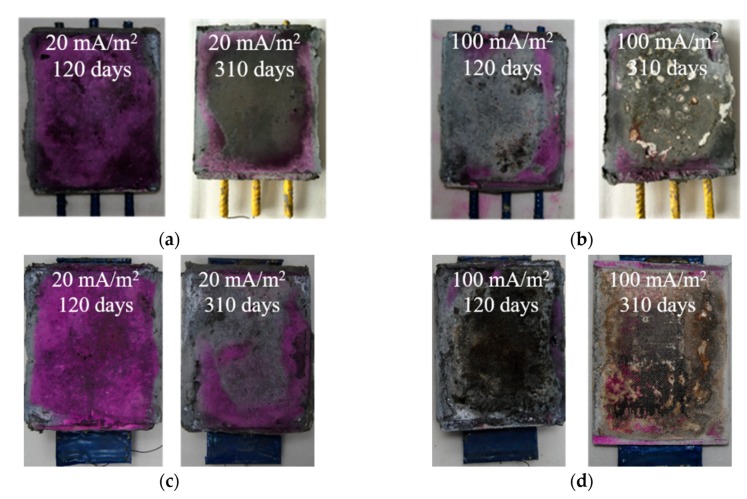
Degradation of the anode interfaces in the chloride-contaminated concrete: (**a**) B-EB-20 specimens; (**b**) B-EB-100 specimens; (**c**) P-EB-20 specimens; (**d**) P-EB-100 specimens.

A 5% phenolphthalein indicator was sprayed onto the anode interface at the concrete surface. Figure 8a shows that the color changed from red to colorless in the B-EB-20 specimens as the EC test time increased. In the B-EB-100 specimens, the surface was colorless after spraying the indicator for the EC tests at both 120 days and 310 days, as shown in Figure 8b. It was confirmed that the anode interface was acidified due to the increasing current density and testing time. Figure 8c,d show similar changes for the P-EB-20 and P-EB-100 specimens. For the specimens with IB CFRP, the color change was the same as that for EB CFRP. It seems that the acidification of the anode interface at the concrete surface is not influenced by the bonding methods of CFRP or the shape of the steel embedded in the concrete; the acidification depends on the scale of the electric charge reaction at the anode interface.

#### 3.3.2. Degradation of CFRP

**Figure 9:**
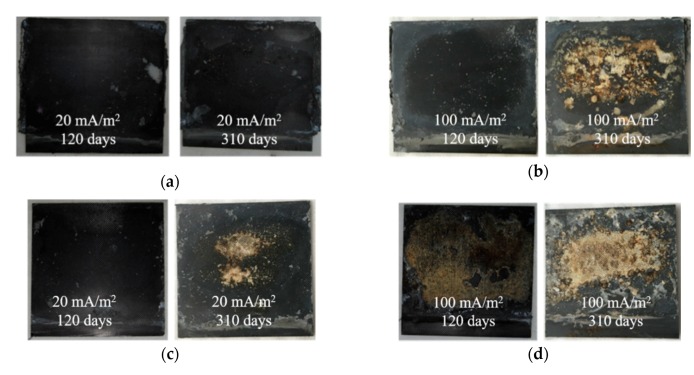
Degradation of the EB and IB CFRP: (**a**) B-EB-20 specimens; (**b**) B-EB-100 specimens; (**c**) B-IB-20 specimens; (**d**) B-IB-100 specimens.

**Figure 10:**
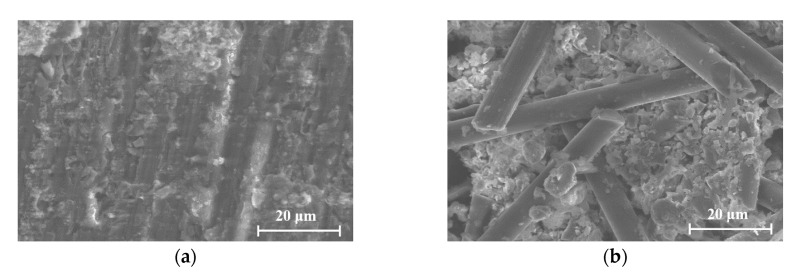
Scanning electron microscopy results of the CFRP after EC test for 310 days: (**a**) B-EB-20 specimens; (**b**) B-IB-100 specimens.

Figure 9 shows the appearance of the CFRP at the bonding surface with concrete. After exposure to anodic polarization, the change that occurred at the CFRP surface was influenced by not only the applied current density and polarization time but also the CFRP bonding method. Regarding the influence of the bonding method, Figure 9 shows that the degradation in the EB CFRP (Figure 9a,b) is inferior to that in the IB CFRP (Figure 9c,d) when the applied current density and polarization time were identical. A possible explanation for this finding is that the EB CFRP was bonded to concrete through a conductive adhesive binder mixed with short-chopped carbon fibers, indicating that the zone to be polarized was enlarged, which reduced the degradation at the surface of the CFRP. Compared to the EB CFRP, the polarization effect on the IB CFRP was probably intense because the IB CFRP was embedded into the concrete during mixing. To investigate the influence of current density and polarization time, it is evident that more degradation could be observed at a current density of 100 mA/m^2^ than at 20 mA/m^2^ under the same polarization conditions. Similarly, more degradation was observed with increasing polarization time when the current density was applied at the same level, and the degradation observed in the CFRP was more evident as the current density increased at specified test time. These performance trends were identical when the steel rebars and steel plate were embedded in the concrete. Obviously, the degradation of CFRP during exposure to anodic polarization was largely influenced by the charge density (i.e., current density times test time) and CFRP bonding method. Microscopic analysis of the CFRP is shown in Figure 10. The carbon fiber reinforcement in the CFRP was solidified with epoxy resin in the B-EB-20 specimen, as shown in Figure 10a. Substantial damage was observed in the epoxy resin and carbon fiber reinforcement in the B-IB-100 specimen, as shown in Figure 10b. These findings confirm that more degradation was induced as the charge density increased.

**Figure 11:**
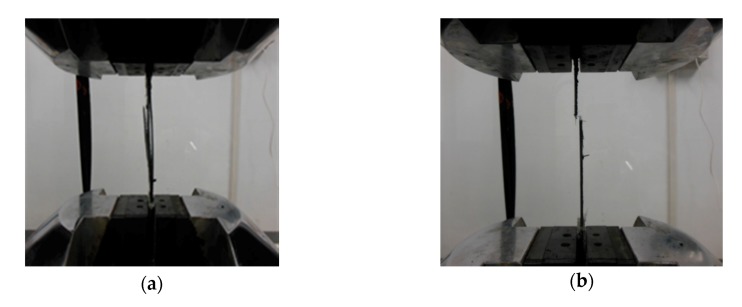
Failure modes of the CFRP after EC test for 310 days: (**a**) Delamination failure; (**b**) Rupture failure.

Such degradation probably caused a decrease in the mechanical properties of the CFRP, which diminished the behavior of the CFRP as a strengthening material. A series of tensile tests were conducted on CFRP coupons. Two failure modes were observed, as shown in Figure 11. The testing results showed that all the IB CFRP coupons exhibited delamination failure within the gauge length of the coupons, as shown in Figure 11a, whereas the EB CFRP exhibited rupture failure throughout the coupon cross-sections, as shown in Figure 11b. The different degradation mechanisms between EB CFRP and IB CFRP accounts for the change in failure modes under tensile loading.

**Figure 12:**
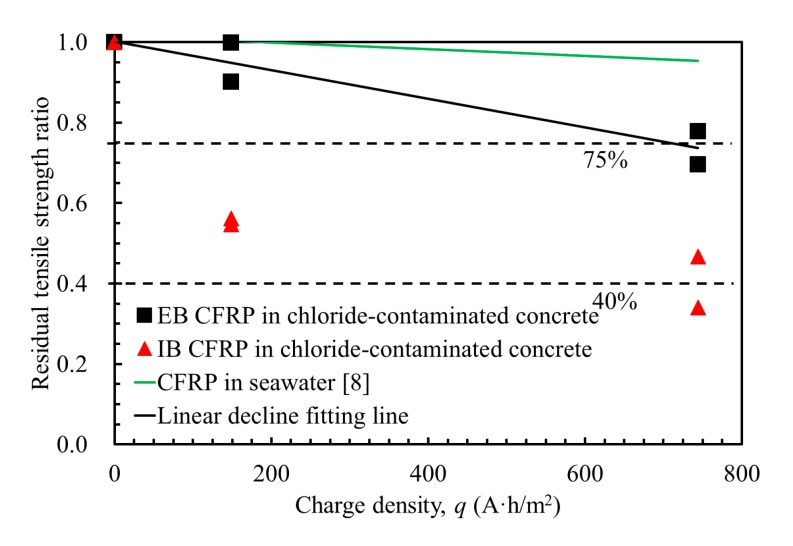
Residual tensile strength of the CFRP.

The tensile load was recorded, and the corresponding residual tensile strength was calculated by dividing the tensile load by the nominal cross-sectional area of the CFRP coupon. The residual tensile strength ratios were calculated by dividing the residual tensile strengths by the tensile strength of an unconditioned CFRP. The average tensile strength results of the CFRP and the corresponding residual tensile strength ratios are presented in Table 3. Compared with the tensile strength of an unconditioned CFRP (*f*_u_RF_ = 968 MPa), the residual tensile strength decreased as the charge density increased for both the IB CFRP and EB CFRP coupons. However, the IB CFRP exhibited a substantially greater decrease in residual tensile strength than that of the EB CFRP at the same current density. Figure 12 shows the residual tensile strength ratio with respect to the charge density. The residual tensile strength ratio decreased linearly in the EB CFRP as the charge density increased, where the residual tensile strength ratio was maintained at 90% when the charge density was 148.8 A·h/m^2^ and reduced to 70% when the charge density increased to 744 A·h/m^2^. For the IB CFRP, the residual tensile strength ratio suddenly dropped to 55% at a charge density of 148.8 A·h/m^2^ and was only 34% at the end of the EC tests in this study. The post-mechanical performance of the EB CFRP was superior to that of the IB CFRP. Moreover, previous investigations [24,25,26] indicated that the usage ratios of CFRP as a strengthening material normally ranged from 40% to 75%. Therefore, the residual tensile strength of the considered CFRP exposed to anodic polarization could still satisfy the basic requirement for strengthening [27,28]. However, the effectiveness of structural strengthening depends on not only the strength of the strengthening material itself but also the bond strength between the strengthening material and concrete. The influence of polarization in ICCP on the bond performance should be further investigated by using single-lap [29] or double-lap shear testing [30] methods.

### 3.4. Prediction of Residual Tensile Strength of CFRP

Based on the above discussion, it was found that polarization in ICCP had a larger influence on the mechanical degradation of the IB CFRP than on that of the EB CFRP. Therefore, it is recommended to adopt the EB bonding method in the application of chloride-contaminated concrete using the ICCP-SS intervention system with dual-functional CFRP materials.

To assess the mechanical behavior of CFRP as a dual-functional material in ICCP-SS system, a prediction of the residual tensile strength of dual-functional EB CFRP was proposed based on the tensile test results, as shown in Figure 12. A linear decline model describing the degradation induced by polarization in ICCP was achieved in Equations (9) and (10). The accumulated charge densities were considered in this model. A similar model was also proposed in a previous study in which the CFRP was polarized in a simulated seawater solution [8], as shown in Equation (11) and Figure 12. Although the chloride content in the current study (1.94% by the weight of water) is identical to that in the study by [8] at a charge density of 744 A·h/m^2^, as shown in Figure 12, the predicted residual tensile strength ratios of the chloride-contaminated concrete with CFRP in simulated seawater solution are 0.70 and 0.93, respectively. This evidence indicates that the degradation of CFRP is more substantial in chloride-contaminated concrete than in a simulated seawater solution, possibly because the interface between the CFRP and concrete was complex and uneven, leading to pitting degradation of the CFRP, whereas the interface of the CFRP in the solution was stable and uniform, causing general degradation.

(9) $$f_{u}=f(q)f_{u\_\mathrm{RF}},$$

(10) $$f(q)=-4.0\times 10^{-4}\times q+1\;0\leq q\leq 744\;A\cdot {h/m}^{2},$$

(11) $$f(q)=-0.856\times 10^{-4}\times q+1\;0\leq q\leq 4224\;A\cdot {h/m}^{2},$$

where *f*_u_ is the residual tensile strength of the CFRP after EC tests, *f*_u_RF_ is the tensile strength of a reference CFRP, and *q* is the charge density during EC tests.

### 3.5. Discussion on the Long-Term Performance of CFRP as a Dual-Functional Material in ICCP-SS

Accelerated tests to assess the long-term performance of CFRP in ICCP-SS system of concrete structures provide general information quickly. Chang et al. [31] proposed converting the accelerated condition to a practical condition by the principle of equal charge quantity, wherein if the total charge quantities in the accelerated and practical conditions are the same, it was assumed that the polarization effects are identical. This relationship has been adopted in many studies on accelerated tests [32,33]. Recently, Zhang et al. [34] reported that the current density has substantial effects on anode material degradation. Therefore, a non-linear conversion model between an accelerated test and a normal operation condition was proposed. Zhang et al. [34,35] concluded that using the principle of equal charge quantity in the accelerated tests will result in the prediction of more severe degradation than that observed under normal conditions.

Therefore, the principle of equal charge quantity is a conservative assessment of the service period of CFRP. The most severe condition in this paper is a current density of 100 mA/m^2^ is applied for 310 days. If a current density of 2 mA/m^2^ is applied, which is the maximum specified applied current density of cathodic prevention of RC structures [36], the corresponding service period of CFRP in ICCP-SS is more than 42.5 years. During this period of 42.5 years, the steel in chloride-contaminated concrete could be protected against corrosion, and the residual tensile strength of the CFRP could retain more than 70% of its original tensile strength, which satisfies the general strength requirement for strengthening concrete [24,25,26]. Further investigations on the bonding degradation of the CFRP/concrete interface suffered to polarization in ICCP are still necessary.

## 4. Conclusions

The anodic and mechanical behavior of CFRP as a dual-functional material in chloride-contaminated concrete was assessed and discussed through the EC tests followed by the tensile tests. The conclusions are as follows:

(1) CFRP can be efficiently used as anode in ICCP which results in the reduction of corrosion of steel reinforcement in chloride-contaminated concrete.
(2) The degradation of anodic behavior of CFRP induced by polarization in ICCP could be presented by the driving voltages.
(3) The residual tensile strength of the CFRP decreased as the charge density increased, but the sufficient residual tensile strength proven that the mechanical behavior of CFRP suffered to polarization in ICCP is acceptable.
(4) To adopt EB CFRP at a current density no greater than 20 mA/m^2^ is recommended for ICCP-SS intervention in chloride-contaminated concrete.
(5) The service period of CFRP in ICCP-SS system could be longer than 42.5 years under the maximum specified applied current density of cathodic prevention for concrete structures.

## Figures and Tables

**Table 1:** Mix proportions of the chloride-contaminated concrete mixture.

Cement (kg/m^3^)	Simulated Seawater (kg/m^3^)	Standard Quartz Sand Aggregate (kg/m^3^)
594	220	1188

**Table 2:** Particle size distribution of the standard quartz sand [10].

Square Mesh Size (mm)	Cumulative Sieve Residue (%)
2.00	0
1.60	7 ± 5
1.00	33 ± 5
0.50	67 ± 5
0.16	87 ± 5
0.08	99 ± 1

**Table 3:** Test specimen parameters and tensile test results.

Specimens	Shapes of Embedded Steel	CFRP Bonding Methods	Current Density (mA/m^2^)	Residual Tensile Strength, *f*_u_ (MPa)	Residual Tensile Ratio (*f*_u_/*f*_u_RF_) ^1^	Failure Modes
RF1	Rebar	NA ^2^	NA	NA	NA	NA
RF2	Plate	NA	NA	NA	NA	NA
RF3	Rebar	NA	NA	NA	NA	NA
RF4	Plate	NA	NA	NA	NA	NA
B-IB-20	Rebar	Internally	20	530	0.55	Delamination
B-IB-100	Rebar	Internally	100	452	0.47	Delamination
P-IB-20	Plate	Internally	20	544	0.56	Delamination
P-IB-100	Plate	Internally	100	330	0.34	Delamination
B-EB-20	Rebar	Externally	20	872	0.90	Rupture
B-EB-100	Rebar	Externally	100	673	0.70	Rupture
P-EB-20	Plate	Externally	20	967	0.99	Rupture
P-EB-100	Plate	Externally	100	753	0.78	Rupture

^1^ The tensile strength for the reference sample of CFRP before EC tests was 968 MPa (*f*_u_RF_); ^2^ NA = not applicable.
